# The versatility of evolutionary intelligent tri‐objective treatment planning for cervical cancer brachytherapy

**DOI:** 10.1002/mp.18022

**Published:** 2025-08-11

**Authors:** Leah R. M. Dickhoff, Ellen M. Kerkhof, Heloisa H. Deuzeman, Danique L. J. Barten, Laura A. Velema, Lukas J. A. Stalpers, Bradley R. Pieters, Carien L. Creutzberg, Peter A. N. Bosman, Tanja Alderliesten

**Affiliations:** ^1^ Department of Radiation Oncology Leiden University Medical Center Leiden The Netherlands; ^2^ Department of Radiation Oncology Amsterdam University Medical Center Amsterdam The Netherlands; ^3^ Cancer Biology and Immunology Cancer Center Amsterdam Amsterdam The Netherlands; ^4^ Imaging and Biomarkers Cancer Center Amsterdam Amsterdam The Netherlands; ^5^ Evolutionary Intelligence Research Group Centrum Wiskunde & Informatica Amsterdam The Netherlands

**Keywords:** automated treatment planning, cervical cancer brachytherapy, multi‐objective optimization

## Abstract

**Background:**

A multi‐objective automated treatment planning approach, called BRIGHT, has demonstrated success in prostate cancer brachytherapy (BT). BRIGHT optimizes directly on dose‐volume metrics, aligning with clinical protocol goals, and produces multiple plans that represent different trade‐offs between tumor coverage and healthy organ sparing. Current automated treatment planning methods either do not optimize directly on dose‐volume metrics or generate a single plan, which is only considered optimal in the specific optimization model.

**Purpose:**

We extended BRIGHT to cervical cancer BT, for which adding a third objective to the existing bi‐objective approach was deemed necessary. In this work, we present the algorithmic adaptations made to the approach and highlight its flexibility, which enables straightforward inclusion of customizations. We further demonstrate that this approach produces clinically acceptable plans.

**Methods:**

The first two objectives in the proposed approach pertain to the EMBRACE‐II protocol, which is divided into tumor coverage and healthy organ sparing. The third objective encompasses added aims, which were deemed necessary to be included to ensure dose distribution shape characteristics not captured in the EMBRACE‐II protocol but which can also readily be tuned to include local clinical preferences. We illustrate this by proposing four different customizations: a baseline customization and three different customizations that lead to (potentially distinct) pear‐shaped dose distributions, often desired in cervical cancer BT. We include optimization with contiguous volumes, a capability distinctive to BRIGHT, as an option for dose distribution shape optimization. We tested all four customizations on 269 BT fractions (123 patients), and studied differences in runtimes, 3D dose distributions, as well as obtained dose‐volume values. Clinical acceptability was evaluated for six representative patient cases, by presenting the resulting set of plans for all customizations to a BT team of two radiation oncologists, a medical physicist, and a radiation therapy technologist. They were asked to assess whether there is at least one acceptable plan per patient in the given set of plans.

**Results:**

Treatment plans can be generated in under 2.8 min with the baseline tri‐objective BRIGHT, or 3.7 min if contiguous volumes are included, even though 260.000 dose calculation points are used for highly accurate dose estimation during optimization. There are visual differences in dose distributions for some of the six patient cases when using the distinct customizations, although generally pear‐shaped distributions were obtained. The contiguity of the dose distributions resulting from optimizing with contiguous volumes can be advantageous in special cases where the high‐dose region is preferred in the target area, as well as directly being tied to the location of the inserted applicator. Achieved dose‐volume values are clinically comparable between all four customizations. The BT team indicated that 3/4 customizations included at least one clinically acceptable plan for all six patients.

**Conclusions:**

Clinically acceptable plans for cervical cancer BT can be quickly generated using the new tri‐objective version of BRIGHT. This approach allows for straightforward customization to accommodate local clinical preferences. We demonstrated this versatility through various customizations that produced generally pear‐shaped, yet potentially distinct, dose distributions, with comparable dose‐volume values according to the EMBRACE‐II protocol.

## 1 INTRODUCTION

Radiation treatment planning tackles the complex question of how to deliver enough dose to the tumor (i.e., target volume), while limiting the dose to the healthy organs that surround the tumor. In brachytherapy (BT), the dose is delivered by a radioactive source residing at specific dwell positions for distinct times (called dwell times), where a longer time (with the same source) corresponds to a higher delivered dose. Treatment planning thus comprises optimizing a set of dwell times associated with the possible dwell positions. In cervical cancer BT, the treatment is given through the use of an intracavitary applicator (typically consisting of an intrauterine part and two ovoids), and, in most cases, additional interstitial needles in the parametrial tissues. The generated BT treatment plan is evaluated based on dose‐volume (DV) metrics, as well as a visual inspection of the 3D dose distribution.

Recent advancements have demonstrated the superiority of automated treatment planning methods over conventional manual planning, both in terms of planning time and quality.^1^, ^2^, ^3^ Even though DV metrics present the main quantifiable treatment plan evaluation criteria, most automated methods do not directly optimize on them, since their exact definitions comprise non‐continuous, non‐differentiable, and non‐convex functions. These cannot be solved by gradient‐based optimizers that are used by most automated treatment planning methods for their speed advantages, which is why they simplify the problem by making it smooth and convex.^4^, ^5^, ^6^, ^7^

Algorithms which can tackle optimizing directly on the DV metrics are evolutionary algorithms (EAs), since they do not depend on gradients. EAs are widely considered a subfield of AI.^8^ Similar to natural evolution, EAs iteratively select better solutions and generate variations thereof to form new candidate solutions. Although classic EAs may pose a problem of being prohibitively slow, model‐based EAs that learn and exploit problem structure during optimization offer means to overcome this. In particular, BRIGHT (BRachytherapy via artificially Intelligent GOMEA‐Heuristic based Treatment planning), using a bi‐objective problem formulation within the Multi‐Objective Real‐Valued Gene‐pool Optimal Mixing Evolutionary Algorithm (MO‐RV‐GOMEA),^9^ was proven efficient and effective for BT treatment planning. Its ability to be GPU‐parallelized has allowed for speedups such that high precision treatment plans can be obtained in under 3 min (or under 30s when using less precise dose calculations).^10^ BRIGHT presents the user with multiple plans, which intuitively capture the trade‐off of target coverage versus organ at risk (OAR) sparing. Its implementation for prostate high‐dose‐rate (HDR) BT has been successfully introduced into clinical practice at the Amsterdam University Medical Center.^11^

The multi‐plan output allows for direct insight into the achievability of aims and variation in associated underlying 3D dose distributions. This is especially useful since factors beyond the specified DV metric aims, such as requirements by individual clinics, comorbidity considerations, varying importance of regions within a delineated target, and preferences of radiation oncologists, play a pivotal role in treatment plan evaluation. Since these include non‐quantifiable characteristics, incorporating them into automated planning is challenging. Therefore, it is essential to present the BT team with not just one, but a set of plans to choose from. This is further supported by the fact that in cervical cancer BT, even plans which satisfy all DV aims from the internationally known and recommended EMBRACE‐II protocol,^12^ can be deemed as clinically unacceptable, because of undesirable properties in the dose distributions.^13^

In this work, we introduce an adaptation of BRIGHT that enables straightforward customization to meet the specific needs of individual clinics. We furthermore apply BRIGHT to cervical cancer BT and show how tailoring can be performed.Specifically, we present a tri‐objective version of BRIGHT, in which the first two objectives relate to DV aims from the EMBRACE‐II protocol^12^ pertaining to target coverage and EMBRACE‐II DV aims concerning OAR sparing. The third objective encompasses additional aims that were judged necessary to ensure certain dose distribution shape characteristics not captured in the quantitative metrics of the EMBRACE‐II protocol. Furthermore, cervical cancer BT differs from prostate cancer BT that BRIGHT was originally tested on, for example in its intracavitary nature and larger number of concerned target volumes and OARs. The added aims were tuned in a feedback loop with local BT teams. This tri‐objective approach has already been shown to outperform clinical manual planning in terms of achieved DV values in previous work^14^ on a subset (n=81, with stricter inclusion criteria) of the cases used in this paper. Specifically, BRIGHT achieved EMBRACE‐II protocol aims (for at least one plan in the set of plans) in 88.9% of patient cases, compared to 47.2% in clinical practice, although the comparison was made on a per‐fraction basis. Next to DV aims, the EMBRACE‐II protocol defines less strict DV limits,^12^ which were reached in 88.9% (72/81) of cases in the clinic versus 100% (81/81) by BRIGHT. BRIGHT‐generated plans were superior in terms of OAR sparing while maintaining comparable target coverage.

In this paper, we show that this added objective can be readily customized to include preferences from local clinics. We test the tri‐objective BRIGHT retrospectively on an extended dataset of 123 cervical cancer patients (total of 269 fractions).

The proposed approach differs from other automated planning methods by employing a multi‐objective worst‐case optimization model, leveraging an Artificial Intelligence (AI)‐based optimization framework rooted in modern model‐based EAs, and its ability to optimize contiguous volumes, for example, constraining isodose volumes to be contiguous. This latter capability is for instance particularly significant for cervical cancer BT, where a pear‐shaped dose distribution is traditionally considered desirable.^15^, ^16^

Here, we explore various customizations for shaping dose distributions, including introducing a pear‐shaped region of interest (ROI) around the applicator and optimizing the contiguous high‐dose volume. The versatility of our tri‐objective approach is demonstrated through the seamless incorporation of these customizations into the third objective. Clinical acceptability is assessed by presenting the resulting plans to a multidisciplinary team, including two radiation oncologists, a medical physicist, and a radiation therapy technologist.

## 2 BACKGROUND

### 2.1 The pear‐shaped dose distribution

In cervical cancer BT, dose prescription to the A points with the Manchester system was introduced in 1938,^21^ since the dose at the A points stood for the average dose in the paracervical triangle.^22^ With two vaginal ovoids and a central uterine tube, a preset dose could be obtained at the A points,^23^ leading to an overall pear‐shaped dose distribution. Since then, CT‐ and afterwards MRI‐based treatment planning have made it possible to include 3D target volumes in dose prescriptions.^24^, ^25^ Nevertheless, standard loading patterns in the uterine part of the applicator as well as in the ovoids, traditionally and still nowadays, start with a normalization to the A points and thereby describe a pear‐shaped isodose volume.^16^ The dose distribution is then further manually optimized, without losing the overall pear‐shape, and doses to the A points, amongst others, are solely reported.

There are no clinical studies explicitly proving the superiority of pear‐shaped dose distributions over non‐pear‐shaped ones. Rather, this is assumed to be the case due to years of successful treatments, adapting to the anatomy of the cervical tumor with extension in the parametrial tissues (width of target volume) and in the uterus (length of target volume). Therefore, developed automated treatment planning methods often include a means to make the dose distribution pear‐shaped. One of them implemented two regions: a ‘pear’ of 9 mm around the intracavitary applicator, for which $V_{5.9\;\mathrm{Gy}}>95\%$, and a ‘pear‐inside’ of 5 mm around the applicator, for which doses <8Gy get penalized.^1^ Comparably, in another method, a structure defined by two line segments parallel to and on the outer side of the ovoids is used.^26^ Yet a different method necessitates a pear shape that is contoured by hand.^4^ There are however also methods that do not include a pear shape.^27^, ^28^

### 2.2 Dose calculation points

In BRIGHT, DV values are estimated using dose calculation points (DCPs), which are sampled uniformly at random in each of the organs or ROIs. The amount of dose in each point is calculated using the TG‐43 formalism.^17^ These values can then be used to approximate key indicators that are considered to be important. Specifically, volume metrics $V_{d}$ can be calculated by taking the subset of DCPs with which at least a certain dose d is associated. For dose metrics $D_{v}$, the subset of points that are planned to receive the highest dose is taken, such that the volume of this subset is v, after which the value for $D_{v}$ equals the lowest dose associated with any of the points in the subset. Thus, a larger number of DCPs implies a more precise computation of the DV values, at the expense of increased computational cost and thus slower optimization.

### 2.3 Bi‐objective optimization

Bi‐objective BRIGHT for prostate HDR BT is clinically used at the Amsterdam University Medical Center.^11^ BRIGHT can optimize any indicator $I_{j}$ with an associated aim $A_{j}$, by calculating the differences compared to the aims in an objective function o(t) at time t as:

(1) $$o\left(t\right)=\sum_{j\;\in \;\text{indicators}}\left(I_{j}-A_{j}\right)\;\;\;\text{with}\;I,A\in R.$$

The most used indicators in BT are DV metrics. BRIGHT directly optimizes on the DV metrics as given in a clinical protocol by intuitively grouping them into its two objectives called the least coverage index (LCI) and least sparing index (LSI), depending on whether the aim associated with the DV metric should be maximized or minimized. For each DV metric x, the value to optimize is the difference between its current value ${\text{DV}}^{x}$ and its aim ${\text{DV}}_{\text{aim}}^{x}$. In order to compare them, each difference is normalized,^10^ to then be attributed a weight $w_{x}$ that is automatically set every generation of the optimization algorithm embedded in BRIGHT (see section 2.4). Exponentially higher weights are given to larger differences to actively optimize on the currently most violated DV metric mostly, but still optimize on the other DV metrics if the worst case can no longer be improved:^10^

(2) $$\begin{array}{cc} {\mathrm{LCI}}_{w}\left(t\right) & =\sum_{x\;\in \;\text{coverage aims}}w_{x}\left({\text{DV}}^{x}-{\text{DV}}_{\text{aim}}^{x}\right),\\ {\mathrm{LSI}}_{w}\left(t\right) & =\sum_{x\;\in \;\text{sparing aims}}w_{x}\left({\text{DV}}_{\text{aim}}^{x}-{\text{DV}}^{x}\right). \end{array}$$

This formulation implies that optimization is continued even after all aims have been reached, and a positive LCI or LSI implies that all aims associated with its respective DV metrics have been satisfied.

Constraints can also be included in BRIGHT. Hard constraints can be seen as conditions which are never acceptable throughout the optimization, treatment plans which dissatisfy them get a strictly positive constraint value. Examples in cervical cancer BT include a dwell time modulation restriction, limiting by how much dwell times of neighboring dwell positions can differ,^18^ and a maximum contribution of all needles set to 40% with respect to the total applicator and needle contribution (customizable), as well as 20% for each single needle, though both values are adjustable.

Directly using this bi‐objective approach for cervical cancer BT by taking the aims given in the EMBRACE‐II protocol led to clinically unacceptable treatment plans even though all EMBRACE‐II aims would be surpassed.^13^ Therefore, additional aims were defined in an iterative feedback loop with a multi‐disciplinary BT team of Leiden University Medical Center and Amsterdam University Medical Center. All EMBRACE‐II aims as well as added aims (for pre‐existing and newly defined ROIs) are presented in Table 1.

**TABLE 1:** Cervical cancer HDR BT planning criteria.

Volume	Use	Objective	Coverage aims		Sparing aims	Added aims
${\mathrm{CTV}}_{\mathrm{HR}}$	Target	LCI, LSI, LAI	$D_{90\%}>7.8\;\left(90\right)$ Gy	$D_{98\%}>5.8\;\left(75\right)$ Gy	$D_{90\%}<8.3\;\left(95\right)$ Gy	$V_{100\%}>99.9\%$
${\mathrm{CTV}}_{\mathrm{IR}}$	Target	LCI, LAI	$D_{98\%}>3.5\;\left(60\right)$ Gy			$V_{50\%}>99.9\%$
${\mathrm{GTV}}_{\mathrm{res}}$	Target	LCI	$D_{98\%}>8.3\;\left(95\right)$ Gy			
Bladder	OAR	LSI			$D_{2{\mathrm{cm}}^{3}}<5.5\;\left(80\right)$ Gy	
Rectum	OAR	LSI			$D_{2{\mathrm{cm}}^{3}}<4.0\;\left(65\right)$ Gy	
Sigmoid	OAR	LSI			$D_{2{\mathrm{cm}}^{3}}<4.5\;\left(70\right)$ Gy	
Bowel	OAR	LSI			$D_{2{\mathrm{cm}}^{3}}<4.5\;\left(70\right)$ Gy	
Recto‐vaginal point	OAR	LSI			$D_{\text{point}}<4.0\;\left(65\right)$ Gy	
Mid‐${\mathrm{CTV}}_{\mathrm{IR}}$	Target	LAI				$V_{100\%}<25\%$
Core‐${\mathrm{CTV}}_{\mathrm{HR}}$	Target	LAI (optional)				$V_{200\%}>99.5\%$
${\mathrm{Pear}}_{\text{intrauterine}}$	Target	LAI (optional)				$V_{200\%}>99.5\%$
${\mathrm{Pear}}_{\text{ovoids}}$	Target	LAI (optional)				$V_{200\%}>90\%$
Bottom‐normal‐tissue	OAR	LAI				$D_{90\%}<25\%$
Mid‐normal‐tissue	OAR	LAI				$V_{100\%}<0.1\%$
Top‐normal‐tissue	OAR	LAI				$V_{100\%}<0.2\%$

*Note*: Aims: $D_{v}$: dose metric ‐ minimum dose to the most irradiated subvolume v ${\mathrm{cm}}^{3}$; $V_{d}$: volume metric ‐ subvolume which is planned to receive at least dose d Gy; $D_{\text{point}}$: dose at point.

Coverage and sparing aims from the EMBRACE‐II protocol, and added aims, for a single planning‐aim dose in a four fraction schedule in percentages of 7 Gy, and in brackets the total (4 BT fractions + EBRT) EQD2.

Abbreviations: ${\mathrm{CTV}}_{\mathrm{HR}}$, high risk clinical target volume; ${\mathrm{CTV}}_{\mathrm{IR}}$, intermediate risk clinical target volume; ${\mathrm{GTV}}_{\mathrm{res}}$, residual gross tumor volume; LAI, least added index; LCI, least coverage index; LSI, least sparing index.

See Figure S1 for definitions of Mid‐${\mathrm{CTV}}_{\mathrm{IR}}$, Bottom–normal tissue, Mid‐normal‐tissue, Top‐normal tissue; see section 3.3 for definitions of Core‐${\mathrm{CTV}}_{\mathrm{HR}}$, ${\mathrm{Pear}}_{\text{intrauterine}}$, ${\mathrm{Pear}}_{\text{ovoids}}$ and why their inclusion in the LAI is optional.

An adaptation of BRIGHT for cervical cancer BT was developed, in which the extra set of DV metrics was incorporated into LCI and LSI, and their aims were adaptively configured during optimization.^18^ The downsides are that it becomes less directly clear from the objective values whether the EMBRACE‐II aims are met, the interplay between the EMBRACE‐II and the added aims is intricate and needs careful tuning, and the overall runtime increases. For these reasons, we introduce here tri‐objective BRIGHT, that overcomes all these issues.

### 2.4 The evolutionary algorithm in BRIGHT

The implementation of MO‐RV‐GOMEA ^19^ tailored to BT is called BRIGHT. MO‐RV‐GOMEA is an EA and thus maintains a population that contains a set of potential solutions – in the BT case, treatment plans – that undergoes selection and variation: a subset of the population containing the better solutions is used to create new solutions. One such step is called a generation, of which numerous occur in a loop within a single optimization run. Whether one solution is better than another is established through Pareto dominance, which states that one solution dominates another one if it is better in one objective, and at least as good in all other objectives. The best‐so‐far solutions are kept in an elitist archive. Constraints can also be used in that solutions which violate a hard constraint will never dominate solutions which do not, so they are not kept in the elitist archive. In order to select a specific number of best solutions, they need to be ranked. This is done through domination sorting, in which rank 0 is attributed to all non‐dominated solutions, rank 1 is given to all non‐dominated solutions when rank 0 solutions are excluded from the set, and so forth. The best 35% of solutions are selected and divided into clusters in order to then perform variation.

## 3 MATERIALS AND METHODS

### 3.1 Data

Tri‐objective BRIGHT was tested on a dataset of 123 cervical cancer patients. The full planned dose is divided into and delivered in a number of smaller doses, called fractions, given 1‐7 days apart. Multiple fractions per patient imply that the dataset comprises a total of 269 different magnetic resonance imaging (MRI)‐ or (for a second fraction with the same application) computed tomography (CT)‐based treatment planning cases, called ‘patient cases’ in this work. The number of 269 cases amounts to all fractions in which a new clinical plan was made, that is, occasionally, when the CT revealed that no changes are necessary between fractions based on the same implantation, a same plan is given for two fractions, which is therefore only considered once in this work. This is a retrospective study, since all patients have been treated with 3‐4 fractions of 7 Gy (100% prescription dose) HDR in order to meet the EMBRACE‐II aims, at the Leiden University Medical Center. All consecutively included patients have been treated between 2017‐2021, after planning based on the EMBRACE‐II protocol was introduced. The patients' BT treatment was preceded by an external beam radiation treatment (EBRT) of 25 fractions of 1.8 Gy each. The included patients were treated with the Utrecht^TM^ or the Venezia^TM^ advanced gynecological applicator (Elekta, Stockholm, Sweden), with on average 4 (0‐11) needles, and 88 (32‐223) dwell positions, with step size 2.5 mm.

### 3.2 Adding a third objective

#### 3.2.1 Definition

The third objective includes all DV metrics which are not given in the EMBRACE‐II protocol, combined similarly as done for the first two objectives. It thus comprises extra aims, deemed necessary to obtain clinically acceptable solutions by the institution(s) at hand. The added aims as used in this work include widely desirable properties of the dose distribution such as keeping the high‐dose regions within the target volume, but can also be tuned separately for each institution according to local clinical practice. We thus name the third objective the least added index (LAI). This objective can again be comprised of any set of indicators with associated aims as given in Equation (1). Here, only DV metrics are used as added aims, as a surrogate to model spatial characteristics of the dose distribution, see Table 1. The objective value of a solution is therefore obtained by the normalized weighted sum of the difference between the pre‐defined aim value and the currently obtained value for the DV metric, as explained above for the first two objectives:

(3) $$\begin{array}{cc} & {\mathrm{LAI}}_{w}\left(t\right)\\ & =\left\{\begin{array}{cc} \sum_{x\;\in \;\text{added aims}}w_{x}\left({\text{DV}}^{x}-{\text{DV}}_{\text{aim}}^{x}\right) & \text{if}\;x\;\text{is a coverage aim},\\ \sum_{x\;\in \;\text{added aims}}w_{x}\left({\text{DV}}_{\text{aim}}^{x}-{\text{DV}}^{x}\right) & \text{if}\;x\;\text{is a sparing aim}. \end{array}\right. \end{array}$$

The output is a set of plans which generally represents a non‐dominated front in 3D‐space (i.e., a 2‐dimensional manifold), each dimension corresponding to one objective. Plans can then be insightfully picked from this front, once it is clear how preferable (i.e., high in the used problem formulation) EMBRACE‐II‐defined target coverage, EMBRACE‐II‐defined OAR sparing, and values for the extra aims trade off with each other. This is especially advantageous since an easy distinction can be made between achieving the scientific, internationally recommended aims (denoted by a positive first and second objective), and striving for high additional aims that can include local preferences in the third objective.

#### 3.2.2 Epsilon dominance

The classical definition of Pareto dominance in multi‐objective optimization is that a solution a dominates a solution b, symbolized by a≻b, when a is strictly better than b in at least one objective, and at least as good as b in all other objectives. However, this definition does not include the magnitude of differences in the objective values. It may be that in order to achieve improvement in one or more objectives one or more of the other objective values must strongly deteriorate. Depending on the nature of interaction between the objectives, this becomes more likely with an increasing number of objectives. Such ‘steep’ regions along the Pareto front are likely clinically not relevant, as too large a loss needs to be incurred in some objective(s) to achieve a small improvement in (an)other objective(s). This is especially the case for the LAI objective, as it is likely that added aims are not conflicting with protocol aims (e.g., EMBRACE‐II), increasing the chances that large improvements in LAI are correlated with small losses in LCI and LSI (or vice versa). As such, a Pareto approximation set can consist of a set of solutions which are steeply inclined in objective space, and of which the lower ones (in terms of LAI) are not of clinical interest. Leaving all these options for the user to choose from is thus an unnecessary burden. This problem can be tackled by using epsilon dominance,^20^ where $a{≻}_{\varepsilon }b$, when the objective value o(a)+ε is better than o(b) in at least one objective while being at least as good as b in all other objectives. The parameters $\varepsilon_{x}$, $\varepsilon_{y}$, and $\varepsilon_{z}$ in three dimensions, corresponding to the LCI, LSI, and LAI, respectively, can be tuned separately according to the problem‐specific objective functions. For our approach for cervical cancer BT, we empirically found $\left(\varepsilon_{x},\varepsilon_{y},\varepsilon_{z}\right)=\left(0.002,0.002,0.01\right)$ to give the best results. Furthermore, to obtain a similar scaling for all three objectives, a mapping function $y=\tanh(\frac{x-250}{200})$ is applied to the third objective so that its range is (−1,1). The reasoning behind this mapping function can be found in Supplementary Material B.

**FIGURE 1:**
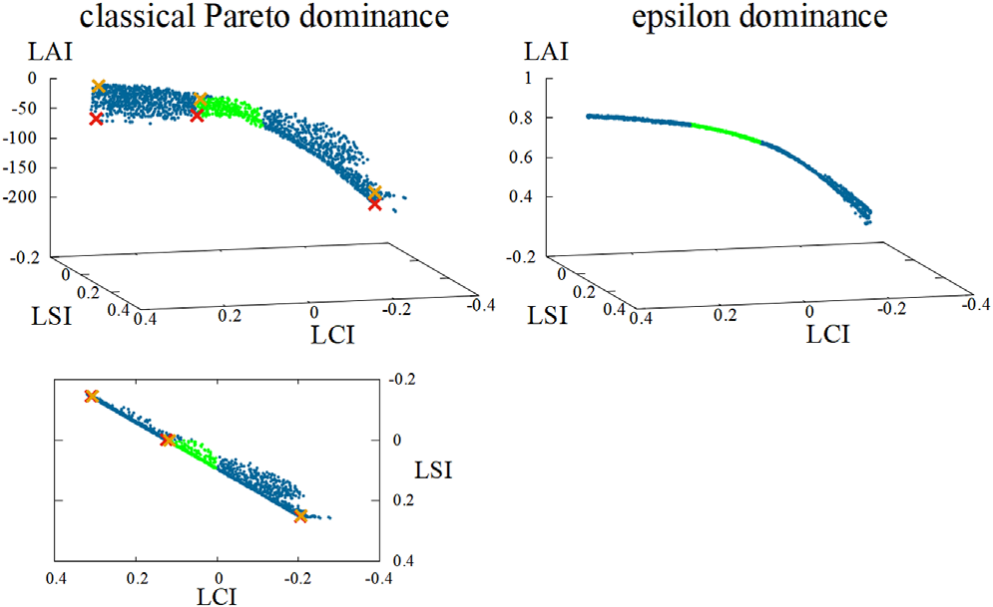
Difference between Pareto approximation fronts resulting from the use of classical Pareto dominance (left) and epsilon dominance with the mapping function for the DV metrics (right). The left bottom figure is the left top figure projected onto the 2D LCI‐LSI plane, with 3 pairs of reference plans marked as orange‐red crosses, to show the steep inclination in objective space. Green: solutions in which all aims from the EMBRACE‐II protocol are achieved (LCI > 0 and LSI > 0); blue: all other solutions. DV, dose‐ volume; LCI, least coverage index; LSI, Least sparing index.

Figure 1 shows a Pareto approximation front resulting from the use of classical Pareto dominance with numerous solutions that are not of clinical interest, as well as a front resulting from the use of epsilon dominance and the mapping function for the DV metrics. The latter has the added advantage that scrolling through the front to inspect the different plans is much more user‐friendly, since plans are essentially in sequential order (in terms of objective values) in one 3D line.

#### 3.2.3 Domination counting

In bi‐objective MO‐RV‐GOMEA, the solutions are ranked through domination sorting to perform selection. In non‐dominated sorting, the best (lowest) rank is assigned to all solutions that are not dominated. The next ranks are iteratively assigned to solutions that are non‐dominated when disregarding solutions of the previous ranks. In three dimensions while using epsilon dominance, this gives rise to the possible problem of domination loops, where three solutions dominate each other, and each pair‐wise domination occurs in another dimension. For instance, it is possible that $a{≻}_{\varepsilon }b$, $b{≻}_{\varepsilon }c$, and $c{≻}_{\varepsilon }a$, since $a_{x}+\varepsilon_{x}≻b_{x}$, $b_{y}+\varepsilon_{y}≻c_{y}$, and $c_{z}+\varepsilon_{z}≻a_{z}$ can be true simultaneously. This issue can be solved by ranking the solutions using domination counting instead, where different ranks are assigned to the solutions based on the number of solutions that dominate them.^30^

### 3.3 Dose distribution shape optimization

We compare two different general approaches, which lead to overall pear‐shaped dose distributions. The first one includes a pear‐shaped ROI, as most other automatic treatment planning methods do, while the second one constitutes a newly proposed optimization approach using one contiguous volume. They are both described in the following subsections.

#### 3.3.1 Pear‐shaped ROI

**FIGURE 2:**
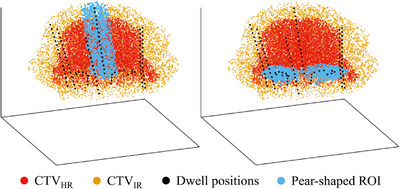
Pear‐shaped ROI (blue) consisting of a 5 mm margin around the intrauterine dwell positions (left) and 5 mm around the ovoid dwell positions (right). Each colored point represents one DCP. DCP, dose calculation point; ROI, region of interest.

The most straightforward way of ensuring a pear‐shaped dose distribution is by adding a pear‐shaped ROI and associating aims with it. This pear consists of a volume around all dwell positions, which are in the ovoids and in the intrauterine part of the applicator, as depicted in Figure 2. Only volume overlapping with the ${\mathrm{CTV}}_{\mathrm{IR}}$ (defined to include the ${\mathrm{CTV}}_{\mathrm{HR}}$) is considered to be part of the pear‐shaped ROI in order not to maximize dose, for example, around the ovoids in rare patient cases where the ovoids are positioned further below the delineated ${\mathrm{CTV}}_{\mathrm{IR}}$. This implies that for a few patient cases, the pear‐shaped ROI only consists of the intrauterine part. The size of the pear can be user‐defined, and, after feedback from our local medical teams, was set to 5 mm around the ovoid and intrauterine dwell positions for all experiments in this work. Optimizing for the 200% isodose line to be around the contours of this pear‐shaped ROI can be directly translated to adding volume metrics of $V_{200\%}^{\text{intrauterine}}>99.9\%$ and $V_{200\%}^{\text{ovoids}}>95\%$ to the third objective (LAI), to directly be optimized on just like the other DV metrics.

#### 3.3.2 Contiguous volumes

##### 3.3.2.1 Definition

The fact that BRIGHT uses DCPs to calculate the values for the different DV metrics (see section 2.2) has the advantage of making optimization for contiguous volumes (contV) possible. To this end, first, a graph is constructed for the DCPs in the ${\mathrm{CTV}}_{\mathrm{IR}}$, where the vertices in the graph are the DCPs. Generating the graph is accomplished by using the graph‐based connected component algorithm called Afforest,^31^ which is a modification of the Shiloach‐Vishkin algorithm.^32^ It has been adapted^33^ and then used in order to connect all the DCPs which are less than a certain distance $l_{contV}$ away from each other. This distance is set such that all adjacent points are connected when the assumption is made that all points represent perfectly cubic volumes uniformly distributed in the ROI:

(4) $$l_{contV}=f\sqrt[{3}]{V_{\text{cc/point}}},$$

where $V_{\text{cc/point}}$ is the volume (in $\;c^{3}\;m$) that each DCP represents inside the ROI and f is a factor of sensitivity which is empirically set to 2.

**FIGURE 3:**
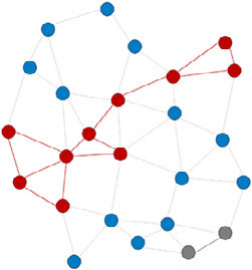
Graph constructed with DCPs as vertices. Red and gray points are points that are planned to receive a dose of at least $d_{contV}$, of which only the red points represent a volume which is larger than a minimum predefined volume (i.e., $v_{contV}$), and therefore constitute the contiguous volume. DCPs, dose calculation points.

Second, all the points which are planned to receive more than a minimum dose of $d_{contV}=250\%$ (of the prescription dose of 7 Gy) are marked. This value indicates that only doses above 250% are included in the contiguous volume. This has been determined in discussion with medical specialists. If two or more points are marked and connected, then their representative volume is calculated. Third, all volumes smaller than a minimum volume $v_{contV}={\left(0.5\;cm\right)}^{3}=0.125$ $\;c^{3}\;m$ are discarded. This is tuned such that volumes smaller than $v_{contV}$, for example, around needle dwell positions, are not considered as an additional contiguous volume. This way, as visualized by the red subgraph in Figure 3, all connected points (which are under $l_{contV}$ away from each other), which are planned to receive a dose of at least $d_{contV}$, and encompass a volume of at least $v_{contV}$, are found.

##### 3.3.2.2 Initialization

**FIGURE 4:**
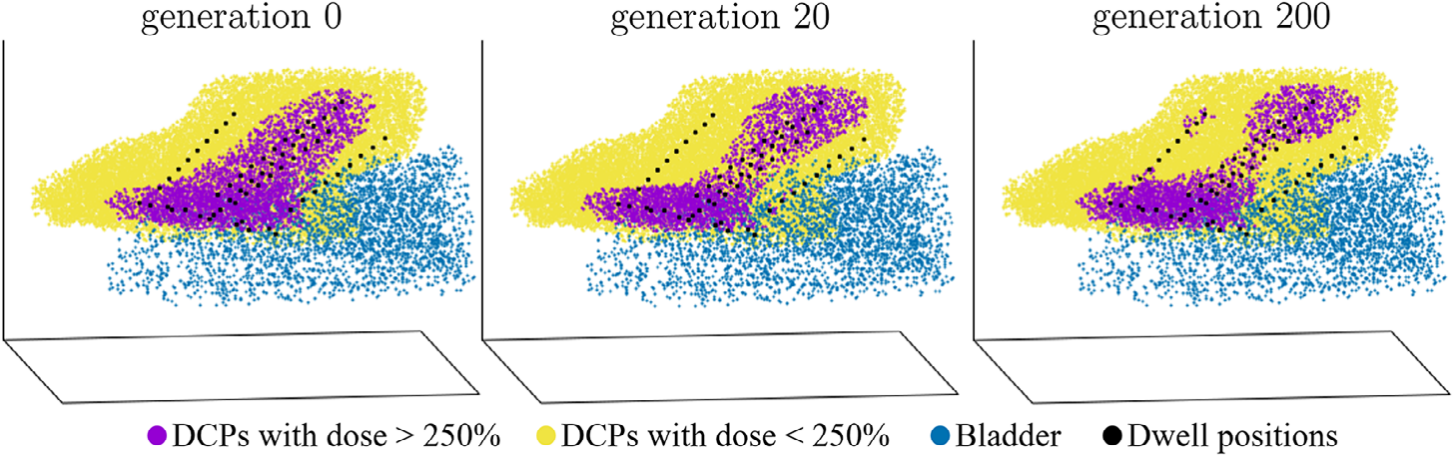
Progression of an optimization run with one contiguous volume (purple). Generation 0, 20, and 200 are shown. Each colored point represents one DCP. Yellow and purple constitute the points in the ${\mathrm{CTV}}_{\mathrm{IR}}$. Purple points represent a dose of more than $d_{contV}=250\%$ and are thus considered for the underlying graph. Volumes smaller than $v_{contV}=0.125$ cm^3^ (e.g., the purple points around the needle visible in the top left at generation 200) are not considered. DCP, dose calculation point.

For cervical cancer BT, the initialization of the dwell times in the applicator is set to values according to dwell weights (defined as the relative dwell time when normalized to 1) of 0.9±0.05, which is equivalent to dwell times of around 9–23 s, depending on the source strength. At the same time, dwell times in the needles are initialized to only [0.5,2.0] s (uniformly distributed). Hence, the initialization reflects a pear‐shaped dose distribution, encompassing one single contiguous volume. Setting a hard optimization constraint to exactly one contiguous volume ensures that no more than one contiguous volume is allowed, thus keeping a contiguous (250%) dose distribution while optimizing for the specific patient at hand. An example of different stages of such an optimization can be seen in Figure 4, in which a contiguous volume is kept through generations 0, 20, and 200, even with an adjacent bladder.

##### 3.3.2.3 Localization

A further option in order to centralize the contiguous 250% isodose volume to the target center is to add a dose aim on the middle region of, for example, the ${\mathrm{CTV}}_{\mathrm{HR}}$, which we call the core‐${\mathrm{CTV}}_{\mathrm{HR}}$. It is defined by the delineated ${\mathrm{CTV}}_{\mathrm{HR}}$ ROI without its outer 6 mm margin (see Figure S1). As such, with a $V_{200\%}>99.5\%$ aim, a higher dose can be strived for in this specific central region, while maintaining the contiguous volume.

##### 3.3.2.4 Runtime considerations

A precise enough representation of contiguous volumes based on the constructed graph, together with the fact that sampling of the dose calculation points is done (uniformly) at random, requires a greater number of points to be sampled in the ${\mathrm{CTV}}_{\mathrm{IR}}$. We empirically found the number of necessary points to be 20.000. This implies that optimizing with contiguous volumes demands slightly more optimization time. We therefore compared necessary runtimes for optimization with and without contiguous volumes; the setup thereof is described in section 3.4.3.

### 3.4 Experiments

We consider multiple customizations of the objectives in order to showcase any possible differences in resulting dose distributions and DV values, as well as the flexibility and potential of BRIGHT to perform optimization for any of these customizations. The following customizations have been considered:

1. Tri‐objective approach without explicit pear shape,
2. In addition to (A): aim on the pear‐shaped ROI ($V_{200\%}>99.5\%$ for intrauterine part, $V_{200\%}>90\%$ around ovoids),
3. In addition to (A): one 250% contiguous volume,
4. In addition to (C): aim on the core‐${\mathrm{CTV}}_{\mathrm{HR}}$ ($V_{200\%}>99.5\%$).

Note that the options are naturally further tunable and extensible and thereby entirely customizable according to a user's wishes. The above selection serves as an example of different possible optimization formulations, while still being interesting in its own right.

#### 3.4.1 DV metric values

The four customization options are first evaluated by placing obtained DV values side by side by means of boxplots over all 269 patient cases. Following clinical practice, values are compared in total equivalent dose in 2 Gy fractions (EQD2, with ${\left(\alpha /\beta \right)}_{\text{targets}}=10$ Gy, ${\left(\alpha /\beta \right)}_{\text{OARs}}=3$ Gy), summed as the given EBRT and planned BT dose over four fractions. The boxplots are obtained by, from the set of generated plans, picking the plan p with the most equally balanced coverage‐sparing trade‐off value, meaning that the worst of both is maximized:

(5) $$p=\underset{p\in plans}{\mathrm{argmax}}\left[\min\left(\text{LCI}(p),\text{LSI}(p)\right)\right].$$

A Wilcoxon signed rank test with α=0.05 is performed to test for significant differences between customizations in DV values per patient case. It is Holm–Bonferroni‐corrected for multiple testing^34^ since one test for every DV metric is carried out. Customizations (B), (C), and (D) are each individually compared to the baseline customization (A).

#### 3.4.2 Dose distributions

Second, the four customization options are assessed by visually comparing 3D dose distributions of six patient cases for which these visual differences are the most noteworthy. For these six cases, the plans resulting from each customization are presented to a BT team of two radiation oncologists, a medical physicist, and a radiation therapy technologist, together, in order to evaluate clinical acceptability. For these six cases, they were asked to peruse the entire set of resulting plans and assess whether there is at least one acceptable plan among them. A self‐developed graphical user interface was used to this end, which allows for navigating the set of plans. For each currently selected plan, the 3D dose distributions are displayed in coronal, sagittal, and axial views, and a table of all obtained DV values is given. The plan according to Equation (5) is shown by default.

#### 3.4.3 Runtimes

Runtimes of the new BRIGHT approach with and without optimizing with contiguous volumes (customization (C) vs. (A)) are compared by analyzing convergence times for both optimizations, where convergence is taken to be when 99% of the difference between ${\mathrm{LCI}}_{10\min}$ and ${\mathrm{LCI}}_{g=1}$ is reached, with ${\mathrm{LCI}}_{10\min}$ being the LCI value after 10 min and ${\mathrm{LCI}}_{g=1}$ the largest LCI value at generation 1. In addition, in at least the subsequent consecutive 20 generations, ΔLCI, the change in the largest found LCI values, should be under $10^{-4}$. Thus, the maximum time t in 30 runs^35^ for every patient case, after which the following two conditions are true, is retained as the convergence time for that approach:^18^

(6) $$\left({\mathrm{LCI}}_{t}-{\mathrm{LCI}}_{g=1}\right)\;/\;\left({\mathrm{LCI}}_{10\min}-{\mathrm{LCI}}_{g=1}\right)>99\%,$$

(7) $$\mathrm{number}\_\mathrm{generations}\_\mathrm{after}\_t(\Delta \mathrm{LCI}<10^{-4})\geq 20.$$

Since the convergence time is hardware‐dependent, the equivalent number of generations is also registered. Note further that the convergence time found for optimization with contiguous volumes (customization (C)) is assumed applicable to all other customization with contiguous volumes, as for instance (D). The same applies to the convergence time found for optimizing without contiguous volumes (tested on customization (A), applicable to (B)).

#### 3.4.4 Comparison with bi‐objective approach

It is essential to compare the previously developed adaptive bi‐objective approach,^18^ within the same software but without the adaptations explained in this article, to the tri‐objective one presented in this work. We do so by comparing obtained DV values from the EMBRACE‐II protocol, since this protocol is internationally used as a plan quality measure. Required runtimes are additionally set side by side by following the steps laid out in section 3.4.3.

#### 3.4.5 Customization selection by a specific institution

In this work, we present four example customizations for the third objective, offering individual institutions the versatility to incorporate local clinical preferences. For a specific institution, these could be readily chosen. In order to showcase how this can easily be achieved, a choice has been made for the Leiden University Medical Center by means of a small clinical evaluation of all four customizations as presented in this work.

#### 3.4.6 Algorithm parameters

The GPU‐parallelized version of BRIGHT^10^ is run on an NVIDIA RTX A6000 GPU for all experiments in this work. In order to speed up optimization even further, a multi‐resolution scheme was implemented that was first introduced for the CPU version of BRIGHT.^36^ This entails that optimization is started on a lower number of dose calculation points. The number of dose calculation points is then increased in a number of $s_{\text{tot}}=4$ steps. With $n_{DCP}$ being the total number of dose calculation points, initial optimization (i.e., step s=1) is done on $2^{-(s_{\text{tot}}-s)}n_{DCP}$ dose calculation points. The total optimization time is thus divided into $s_{\text{tot}}$ parts of equal time, for each of which the number of dose calculation points is determined by increasing s (by 1 per part). Note that for the contiguous volume optimization, the number of dose calculation points in the ROI of which the contiguous volume is computed (${\mathrm{CTV}}_{\mathrm{IR}}$) is kept constant to 20.000.

The total number of dose calculation points (in the last step) is set to 20.000 per ROI, since this number was deemed sufficiently precise for calculations of the DV values in cervical cancer BT during optimization.^18^ Plans are re‐evaluated on 50.000 dose calculation points per ROI, which is the default for the clinical prostate BT implementation following the standard setting in Oncentra Brachy (version 4.5, Elekta AB, Stockholm, Sweden).^10^ The population size is set to 288, the elitist archive maximum size to 1000, and the number of clusters to 12, since these values have been found effective in tri‐objective optimization in BRIGHT.^37^ A total of 30 runs is conducted per patient per customization because of the stochastic nature of BRIGHT. Other user‐definable clinical parameters are related to the deactivation of specific dwell positions and include:

- The number of dwell positions to use in the top of the intrauterine part of the applicator, outside of the ${\mathrm{CTV}}_{\mathrm{IR}}$ (default: 2),
- The minimum distance from the ${\mathrm{CTV}}_{\mathrm{HR}}$ at which needle dwell positions can be used (default: 4.39 mm, derived from clinical plans),
- Whether or not to use the bottom dwell position in the intrauterine applicator (default: no).

## 4 RESULTS

### 4.1 DV metric values

**FIGURE 5:**
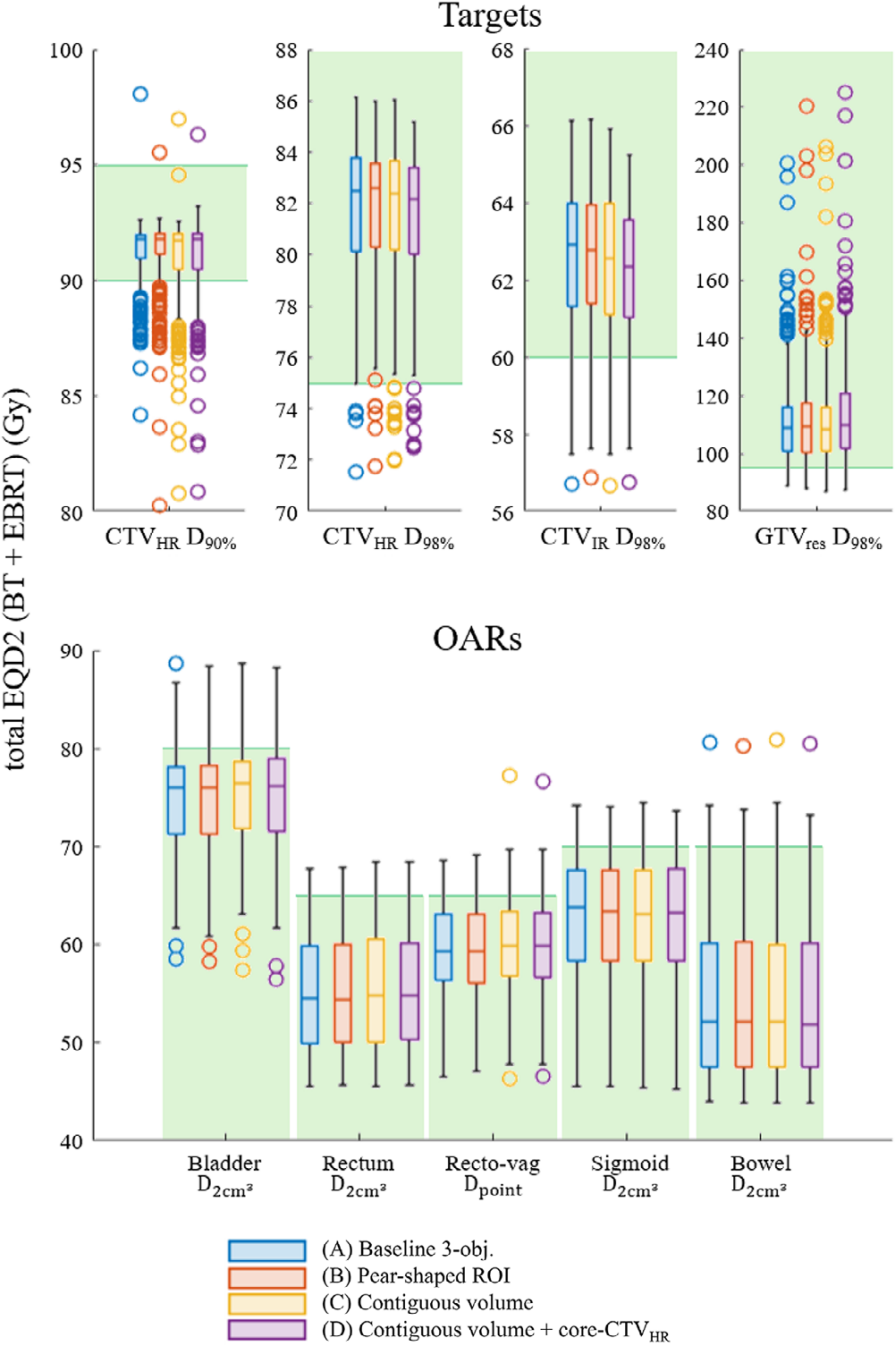
Boxplots of obtained total EQD2 values for the DV metric aims from the EMBRACE‐II protocol when optimizing for the four different customizations, over all 269 patient cases. The green background indicates the area in which all aims included in the EMBRACE‐II protocol are satisfied. Horizontal lines in each box represent the median, and the lower and upper quartiles, the whiskers include data which is ≤1.5× interquartile range away from the bottom/top of the box, and circles denote outliers. The boxplots are based on the median result per patient case calculated over 30 runs. The plan with the most equally balanced coverage‐sparing trade‐off according to Equation (5) is chosen from each set of plans. DV, dose‐volume

Obtained DV values over all patient cases were compared between the different customizations. To this end, boxplots are presented in Figure 5. For every case, 30 runs were performed, and the median DV value is taken over all runs from the one plan (per run) with the most equally balanced coverage‐sparing trade‐off value (Equation (5)). The boxplots are based on these median results for every patient case calculated over 30 runs. Variations of objective values and DV metric values between different runs and patients can be found in Figures S3, S4. Statistics on the contribution of specific catheters can be found in Table S1. The green lines and backgrounds in the boxplots reflect the minimum (for targets) and maximum (for OARs) dose aims as laid out in the EMBRACE‐II protocol. For the patient cases for which these aims were not (all) satisfied, the applicator location with respect to the tumor and surrounding OARs was too challenging. In all of these cases, the clinical plan did not achieve all EMBRACE‐II aims either, but not vice versa. A dosimetric comparison with the corresponding clinical plans, for which fractions are summed up in EQD2 per patient, can be found in Figure S6. Also noteworthy is that the boxplots solely reflect the one plan with the most balanced trade‐off value, so, in practice, for most cases, a plan satisfying a specific aim can still be chosen from the set of plans. The ${\mathrm{CTV}}_{\mathrm{HR}}$ $D_{90\%}$ is the most difficult to achieve in terms of coverage aims: in 98.3% of the cases in which not all aims were achieved, the aim for ${\mathrm{CTV}}_{\mathrm{HR}}$ $D_{90\%}$ was also not reached. The highest values for the ${\mathrm{GTV}}_{\mathrm{res}}$ are found for patients with a particularly small ${\mathrm{GTV}}_{\mathrm{res}}$ which is centrally located at the intrauterine part of the applicator.

Regarding the Wilcoxon signed rank test, results are shown in Table 2 and indicate that, as compared to customization (A), for customization (B), significantly different values are obtained for 6/9 DV metrics, while significant differences are found in 9/9 DV metrics for (C) and 8/9 DV metrics for (D). While results differ significantly statistically, total EQD2 values (medians over 30 runs over all patient cases) differ by at most 1.5 Gy for targets and 0.5 Gy for OARs. First, this is within the clinically insignificant range, and secondly, only the plan with the most equally balanced coverage‐sparing trade‐off (Equation (5)) has been selected for this comparison, while a larger range of DV values can be found within the whole set of plans. We therefore conclude that differences in terms of DV metrics between the four investigated customizations are negligible.

**TABLE 2:** Results of Wilcoxon signed rank test (with Holm–Bonferroni‐corrected α values) for pair‐wise comparison of customizations (B), (C), and (D) with customization (A), where ‘yes’ indicates a significant difference, of which the direction can be seen in Figure 5.

DV metric	Customization (B)	Customization (C)	Customization (D)
$D_{90\%}^{{\mathrm{CTV}}_{\mathrm{HR}}}$	no (p=0.300, α=0.050)	yes (p=0.000, α=0.030)	yes (p=0.001, α=0.050)
$D_{98\%}^{{\mathrm{CTV}}_{\mathrm{HR}}}$	yes (p=0.000, α=0.006)	yes (p=0.000, α=0.010)	yes (p=0.000, α=0.006)
$D_{98\%}^{{\mathrm{GTV}}_{\mathrm{res}}}$	no (p=0.200, α=0.030)	yes (p=0.000, α=0.020)	yes (p=0.000, α=0.007)
$D_{98\%}^{{\mathrm{CTV}}_{\mathrm{IR}}}$	yes (p=0.006, α=0.010)	yes (p=0.000, α=0.007)	yes (p=0.000, α=0.006)
$D_{2cc}^{\mathrm{Bladder}}$	yes (p=0.006, α=0.010)	yes (p=0.000, α=0.006)	yes (p=0.000, α=0.008)
$D_{2cc}^{\mathrm{Rectum}}$	no (p=0.020, α=0.020)	yes (p=0.000, α=0.008)	yes (p=0.000, α=0.010)
$D_{\mathrm{point}}^{\mathrm{Recto}-\mathrm{vag}}$	yes (p=0.001, α=0.007)	yes (p=0.000, α=0.006)	yes (p=0.000, α=0.010)
$D_{2cc}^{\mathrm{Sigmoid}}$	yes (p=0.000, α=0.006)	yes (p=0.000, α=0.010)	yes (p=0.000, α=0.030)
$D_{2cc}^{\mathrm{Bowel}}$	yes (p=0.005, α=0.008)	yes (p=0.010, α=0.050)	yes (p=0.000, α=0.020)

Abbreviations: ${\mathrm{CTV}}_{\mathrm{HR}}$, high risk clinical target volume; ${\mathrm{CTV}}_{\mathrm{IR}}$, intermediate risk clinical target volume; ${\mathrm{GTV}}_{\mathrm{res}}$, residual gross tumor volume; DV, dose‐volume.

### 4.2 Dose distributions

**FIGURE 6:**
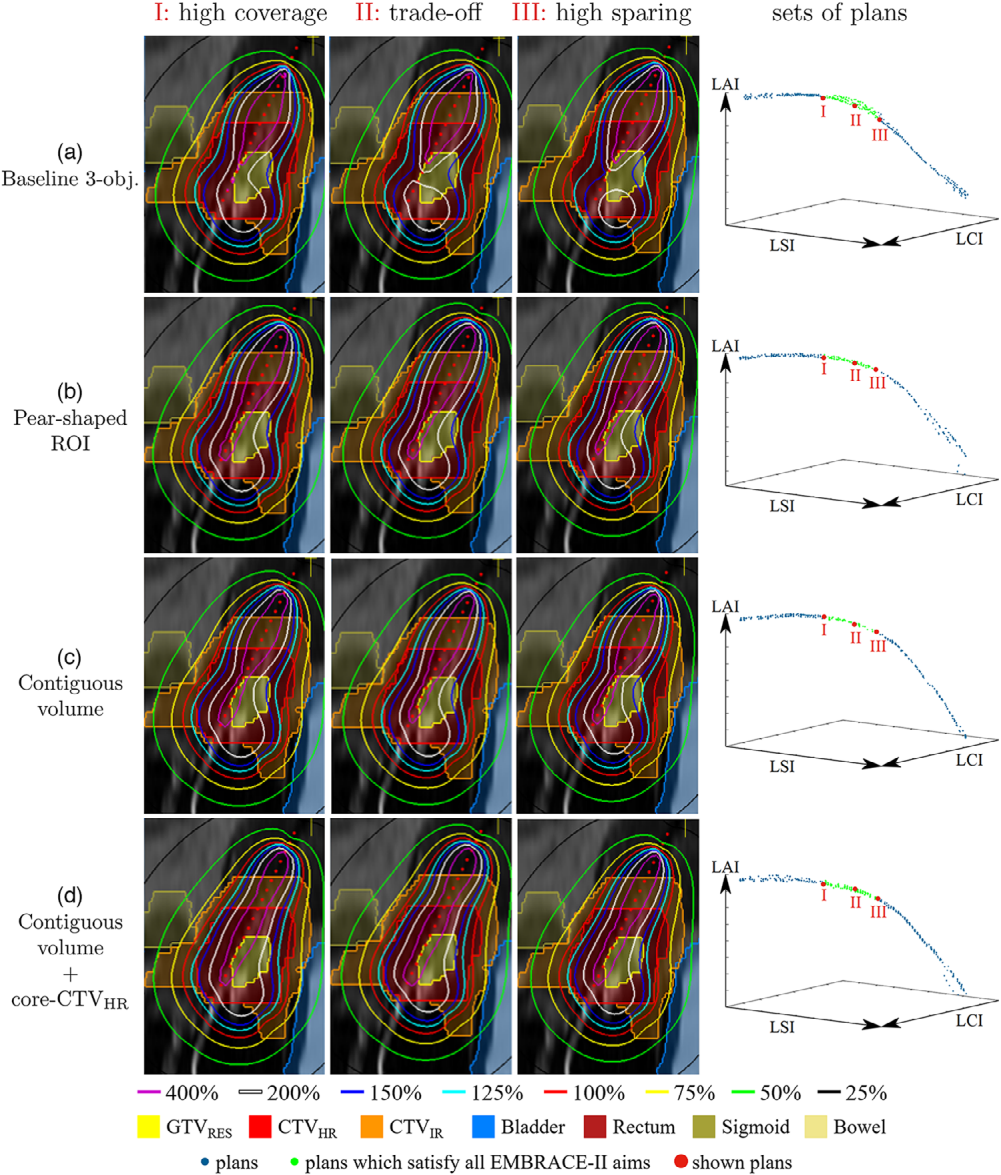
Example of dose distributions resulting from different customizations (rows). Three plans per customization are shown (columns): a high coverage, the most balanced coverage‐sparing trade‐off, and a high sparing plan, which satisfy all EMBRACE‐II aims. These three plans are identified in the Pareto approximation plots on the right, in which the whole generated set of plans is shown, and each axis represents one objective. Sagittal view, visualized with in‐house developed graphical user interface.

Figure 6 shows the dose distributions resulting from the different customization options (as presented in section 3.4) for one representative case of the six evaluated patient cases. Shown plans are automatically selected as depicted on the Pareto approximation fronts on the right side of the figure. The aims from the EMBRACE‐II protocol are achieved for all shown plans. The associated DV values can be found in Table S2, and are similar for all customizations.

For customizations (B), (C), and (D), the BT team judged at least one plan in the set of plans (as depicted on the right in Figure 6) as clinically acceptable for all six patient cases. For (A), in 2/6 cases, all of the plans were assessed to be clinically unacceptable. One can see that, looking at the 3D dose distributions of (A) in Figure 6, the baseline tri‐objective approach (top row), without any explicit pear shape optimization, leads to a separation of the 200% isodose line (i.e., not a single contiguous volume) in an effort to spare the bladder and rectum, which are in close proximity to the ${\mathrm{CTV}}_{\mathrm{IR}}$, as much as possible. This non‐contiguity was judged as not clinically acceptable by the BT team. Note that Figure 4 in which the progression of a contiguous volume optimization can be seen, shows the same patient case. Optimizing with said contiguous volume constraint (row 3 in Figure 6) displays the same characteristics as (A), except with a contiguous volume of 250%, and thereby emulates a pear shape. Adding a core‐${\mathrm{CTV}}_{\mathrm{HR}}\;V_{200\%}$ (row 4) localizes the contiguous volume more towards the center of the target area. Simply including a pear‐shaped ROI (row 2) on the 200% isodose line leads to a more obvious, well‐defined pear shape around the applicator.

**FIGURE 7:**
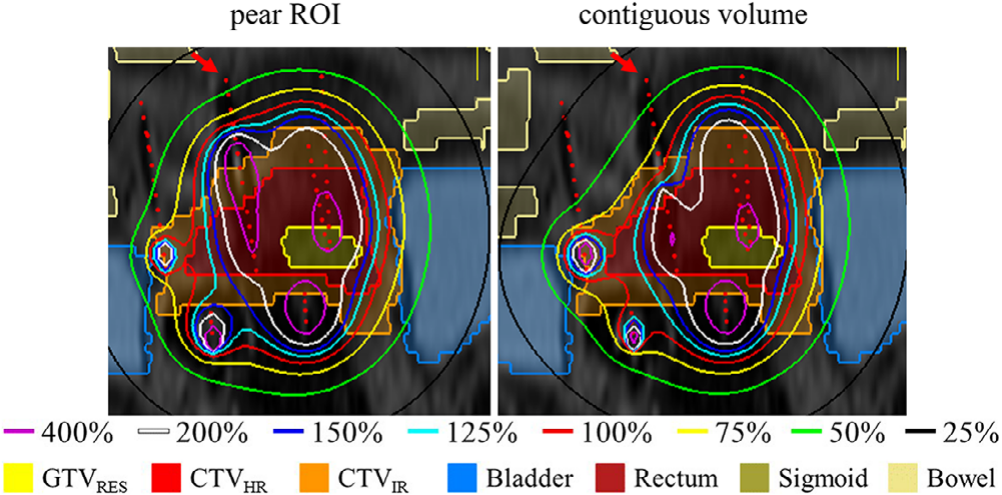
Dose distribution resulting from optimization with pear‐shaped ROI versus contiguous volume for a patient where a needle is used as the intrauterine part of the applicator in terms of dosage. The intrauterine applicator is marked by the red arrow. Coronal view along MRI axes, visualized with in‐house developed graphical user interface. MRI, magnetic resonance imaging; ROI region of interest.

This can, however, also become a drawback of the pear‐shaped ROI, since the localization of the 200% isodose is directly linked to the intrauterine part of the applicator. In patients for whom the tumor location is challenging, for example, if the intracervical and intrauterine canal is not located centrally in the tumor volume, it may well be strongly preferred to use a needle to deliver the dose that would in normal cases be delivered through the intrauterine part of the applicator. Adding a pear‐shaped ROI would, however, still force more dose towards the intrauterine part, whereas optimizing with a contiguous volume enables free placement of the high‐dose region at the target area. This is illustrated in Figure 7, in which isodose lines as high as 400% (of 7 Gy) are given from the upper part of the intrauterine applicator, even though no target volume is delineated there. The plan pertaining to optimization with the contiguous volume was preferred by the BT team over the one resulting from the pear‐shaped ROI inclusion.

It is worth noting that for a large percentage of the patient cases, in which the tumor is fairly centralized, no OARs are directly adjacent to the targets, and the applicator implantation is favorable, all four customization options lead to more similar dose distributions. In these cases, achieving the highest possible DV values already intrinsically leads to a pear shape.

### 4.3 Runtimes

Following the method explained in section 3.4.3, runtimes are examined for both the baseline tri‐objective approach (customization (A) and (B)), and the tri‐objective approach including optimizing contiguous volumes ((C), (D)). The maximum time for the baseline tri‐objective approach is found to be 2.8 min (maximum number of generations is 627), whereas the maximum time for the approach with contiguous volumes is 3.7 min (737 generations).

### 4.4 Comparison with bi‐objective approach

The newly proposed tri‐objective approach outperforms the bi‐objective approach in terms of obtained DV values and above all runtimes. The complete comparison can be found in the Supplementary Material G.

### 4.5 Customization selection by a specific institution

Customization (D) was preferred in all of the six evaluated patient cases. Full details of the setup and results are presented in the Supplementary Material H.

## 5 DISCUSSION

The need for versatility in automated treatment planning in cervical cancer BT stems from the lack of global consensus on what constitutes an ideal treatment plan. Although studies like EMBRACE‐II^12^ provide a strong foundation for standardizing practices, there remain significant inter‐clinic and inter‐country variations in key aspects such as dose distribution shape, dose locality, and the use of interstitial needles or applicators. These differences are likely due to cervical cancer BT, including a large variation in individual tumors, tumor extension, and location of surrounding organs. Given these variations in practices, we believe that automated treatment planning methods should offer customizability to accommodate institution‐specific preferences, at least until a more unified global standard is established.

This study shows that BRIGHT can successfully optimize on different protocols, and by including these differences in the third objective, there is no substantial impact on obtained EMBRACE‐II DV values. This implies that the choice of how to customize the protocol is entirely up to each institution. However, should they desire a readily available protocol, our local clinic has made a selection of a customization (see section 4.5). In order to pick one of the other customizations as presented in this work, it is possible to, for example, directly compare them for a small set of representative patients, as laid out in section 3.4.5. Further specific DV metrics or dose distribution properties can easily be added or removed.

Although this work mostly focuses on differences between practices, BRIGHT's shown flexibility can also be applied to different patient‐specific requirements known before starting BT treatment. For instance, aims can be adjusted or extra aims can readily be added if deemed necessary, for example, due to a challenging tumor extension or tumor location in that patient. Parenthetically, this does not require increasing the runtime, as the same amount of dose calculation points can be used.

Regarding plan navigation and selection, in BRIGHT, a positive LCI or LSI implies that all aims associated with its respective DV metrics have been satisfied. Thus, the key concept is that even if one value dominates an objective – potentially overshadowing other relevant values – a positive LCI or LSI ensures that all these aims are met. If then, a specific requirement for one of the DV metrics still deviates from the standard clinical protocol, it can be adjusted for optimization. The idea is to reduce excessive a posteriori fine‐tuning by enabling a more structured selection process between coverage, sparing, and additional aims within the third objective, while preserving the relative balance between aims.

As to the pear‐shaped dose distribution in cervical cancer BT, we believe that the rationale behind the desirability of the pear shape comes more from the contiguity of the high‐dose region than it does from its location around the applicator. This stems from discussions with medical teams during a multi‐institutional research meeting, and the fact that increased dosage in the cervix and proximal parametria ‐ which is not always centered around the applicator ‐ is considered advantageous because of a higher cell density there.^29^ This is why we implemented an approach which enables this more general goal by optimizing with contiguous volumes.

BRIGHT's evolutionary intelligent non‐linear optimization engine offers considerable potential, particularly in handling complex concepts like these contiguous volumes. While deemed important for evaluation of dose distributions in certain cases, the non‐continuous and non‐differentiable (graph‐based) discrete nature makes typical (gradient‐based) optimizers not suited. This potential of BRIGHT may be leveraged further by practitioners wishing to incorporate yet other non‐continuous descriptors of distribution to optimize.

The runtime necessary to run BRIGHT might seem long, but it is important to note that optimization is done with high‐precision dose calculations, which might not be necessary in clinical practice. Further speed‐up possibilities lie in optimizing the contiguous volume computation, and improving GPU code efficiency. Moreover, the total runtime as currently used, has been determined in a worst‐case manner, that is, based on the patient for which it took the longest for no substantial changes to be detected anymore. For most patients, the golden corner is already reached after 10s and convergence is usually achieved already after 30s. In future work, we also aim to improve on patient‐specific termination criteria. Other cervical cancer automated treatment planning methods^1^ report 4.4–106.4 s, yet just one single plan is computed. Another method^38^ takes 5.8–18.6 s to generate multiple plans, yet the overall planning time including plan navigation and selection is not reported. Time needed for potential manual adjustments of these automated plans are also rarely given. Importantly, both methods use a factor of 10 times less dose calculation points during optimization which is therefore markedly less precise.^1^, ^6^ It is known that this will lead to a considerable deterioration of obtained values for the objectives when doing a re‐evaluation on a large number of dose calculation points as is typically used in a commercial treatment planning system.^10^, ^18^

A current limitation of BRIGHT is that each treatment fraction is optimized independently and for 4 fractions, whereas, in Dutch clinical practice, 3‐4 fractions are delivered to a patient with cumulative dose aims across all fractions. Future work encompasses optimizing fractions incorporating the doses from preceding fractions, as well as giving insights into whether, while planning the first fraction, it could be possible to only use a total of 3 fractions for the specific patient at hand.

Although a numerical comparison with the clinical plans has been done in previous work^14^ and in Figure S6, further research will include a full clinical validation of BRIGHT for cervical cancer BT, by emulating treatment planning with BRIGHT and comparing the selected BRIGHT plan to the clinically used plan. Then, not only DV metric values but also dose distributions and all other clinically relevant plan properties will be included in the comparison.

## 6 CONCLUSIONS

Clinically acceptable optimized treatment plans for cervical cancer BT can be generated within minutes using the new tri‐objective version of BRIGHT. This approach allows for straightforward customization to incorporate individual tumor extension and local clinical preferences without compromising the achievement of official protocol aims. We demonstrated the versatility of the method through several customizations, three of which produced generally pear‐shaped but distinct dose distributions, reflecting different clinical priorities regarding high‐dose regions and their contiguity within the target volume. Importantly, all customizations achieved comparable DV metrics in line with the EMBRACE‐II protocol, ensuring that the choice of customization is left entirely to the medical team, allowing them to tailor treatment planning according to their clinical needs and patient‐specific considerations.

## CONFLICT OF INTEREST STATEMENT

All authors are involved in projects supported by Elekta.

## Supporting information

Supporting Information
