# Supplementary material for: The versatility of evolutionary intelligent tri‐objective treatment planning for cervical cancer brachytherapy
Source: Med Phys. 2025 Aug 11;52(8):e18022. doi: 10.1002/mp.18022 (PMC12340482; doi:10.1002/mp.18022)
Supplement: Supplementary file 1 — Supporting Information [file MP-52-0-s001.pdf]

# Supplementary Material

## The versatility of evolutionary intelligent tri-objective treatment planning for cervical cancer brachytherapy

L.R.M. Dickhoff, E.M. Kerkhof, H.H. Deuzeman, D.L.J. Barten, L.A. Velema, L.J.A. Stalpers, B.R. Pieters, C.L. Creutzberg, P.A.N. Bosman, T. Alderliesten

---

### A. Added aims

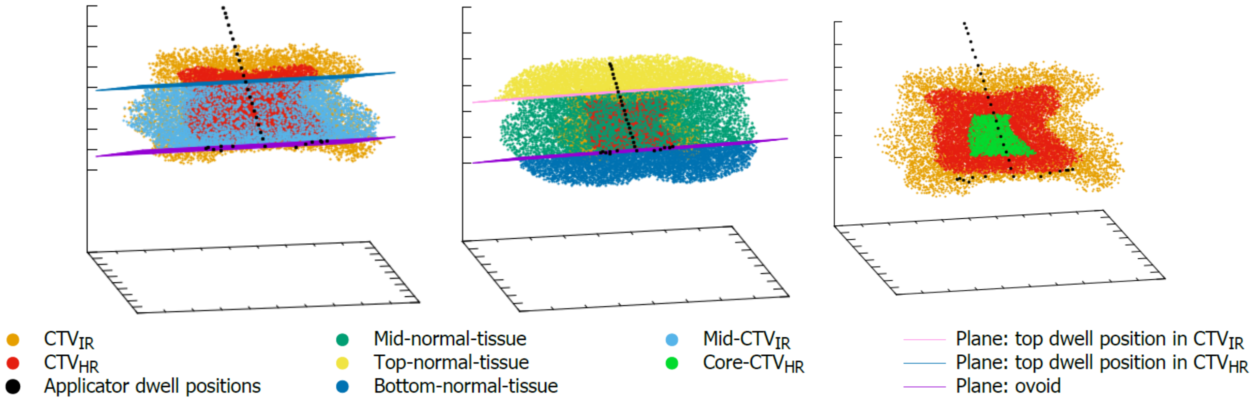


**Supplementary Figure 1:** Added regions of interest: Mid-normal-tissue, Top-normal-tissue, Mid-CTV$_{IR}$, and optional Core-CTV$_{HR}$. Every colored dot is one sampled dose calculation point within the given region of interest. The CTV$_{IR}$, CTV$_{HR}$, and dwell positions in the applicator are plotted as a reference. All visualized planes represent cut-offs of the different added regions and are perpendicular to the intrauterine part of the applicator.

## B. Mapping function for the third objective

A mapping function $y = \tanh(\frac{x-250}{200})$, as visualized in Supplementary Figure 2, is applied to the third objective (LAI) so that its range is $(-1, 1)$. The reasoning behind this is that in the objective functions, the difference between the aim and the current value is normalized for each DV metric. This implies that, if the value of the DV metric is furthest away from its aim, i.e., for the mid-normal-tissue, $V_{100\%} = 100\%$ with target $= 0.1\%$, then the normalized difference to minimize is:

$$\text{difference}_{\text{norm}} = \frac{\text{difference}}{\text{target}} = \frac{V_{100\%} - \text{target}}{\text{target}} = \frac{100\% - 0.1\%}{0.1\%} = \frac{99.9\%}{0.1\%} = 999.$$

Thus, the non-mapped LAI objective function can have values of slightly higher than 999. Most values, however, will be lower than that (as depicted in Figure 1 in the main article, where the top left plot shows LAI values of 0 to 200), since the highest weight is attributed to the most violated DV metric. Thus, a non-linear mapping function was chosen for the LAI. As compared to without mapping, this mapping function caused a change in what DV metric was the most violated only in 0.001% of evaluations, and therefore negligibly impacts the optimization.

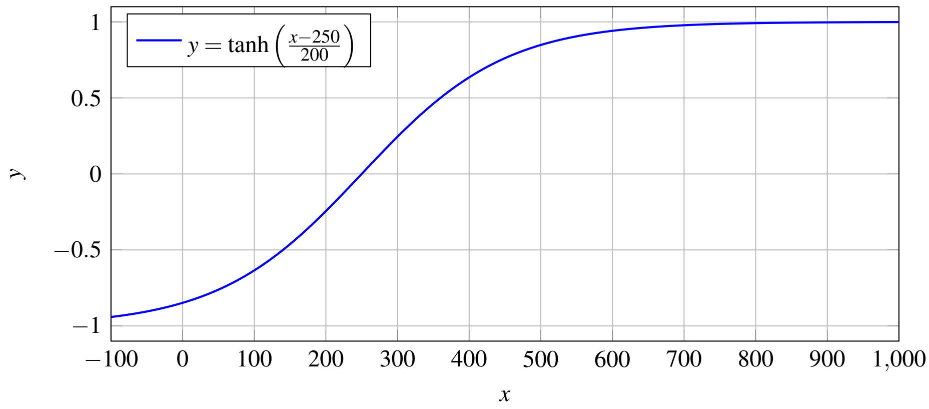


**Supplementary Figure 2:** Mapping function for the third objective (LAI), that maps $x$ to $y$.

## C. Variation of objective values and DV metric values across runs

Kernel density estimates of the distributions of ranges (calculated as the difference between maximum and minimum achieved value over 30 runs) over all patient cases can be found in Supplementary Figure 3 for the LSI and LSI, and Supplementary Figure 4 for the different DV metric values. From each set of plans, the plan according to $p = \text{argmax}_{p \in plans} [\min(\text{LCI}(p), \text{LSI}(p))]$ was taken, which can differ per run even in the worst-case DV metric value which is leading in each objective, resulting in higher ranges between runs. Furthermore, every run samples a different set of dose calculation points (uniformly at random), which leads to a variation of objective values and DV metric values, even for the same plan. Supplementary Figure 5 shows the effect of sampling another set of 500,000 dose calculation points (in total) for the plan $p = \text{argmax}_{p \in plans} [\min(\text{LCI}(p), \text{LSI}(p))]$ of one run per patient case, and then recalculating the DV metric values based on this new set of dose calculation points. In this figure, there is no difference with regards to the recto-vaginal point (kernel density estimate concentrated at 0 for all methods), since this is a defined point which is not altered when sampling new dose calculation points.

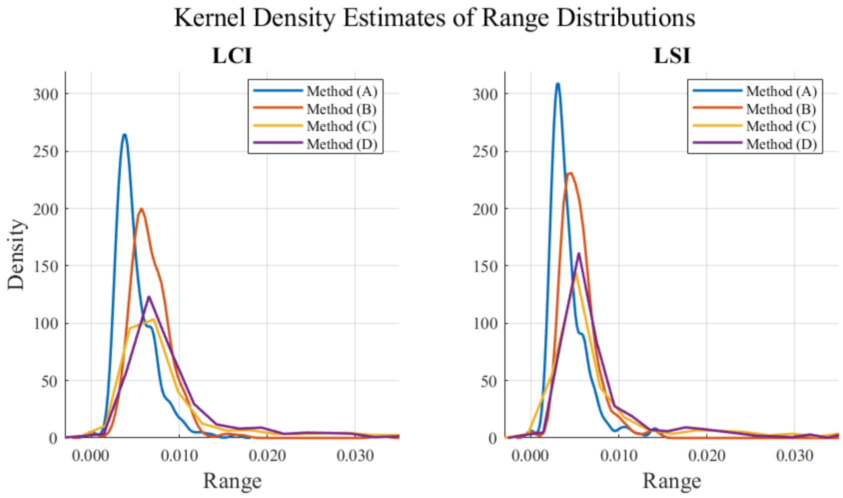


**Supplementary Figure 3:** Ranges (= maximum - minimum achieved value in 30 runs) over all patient cases, plotted as kernel density estimates, separately for the LCI (left) and LSI (right), for the plan according to $p = \text{argmax}_{p \in plans} [\min(\text{LCI}(p), \text{LSI}(p))]$, every color representing one method.

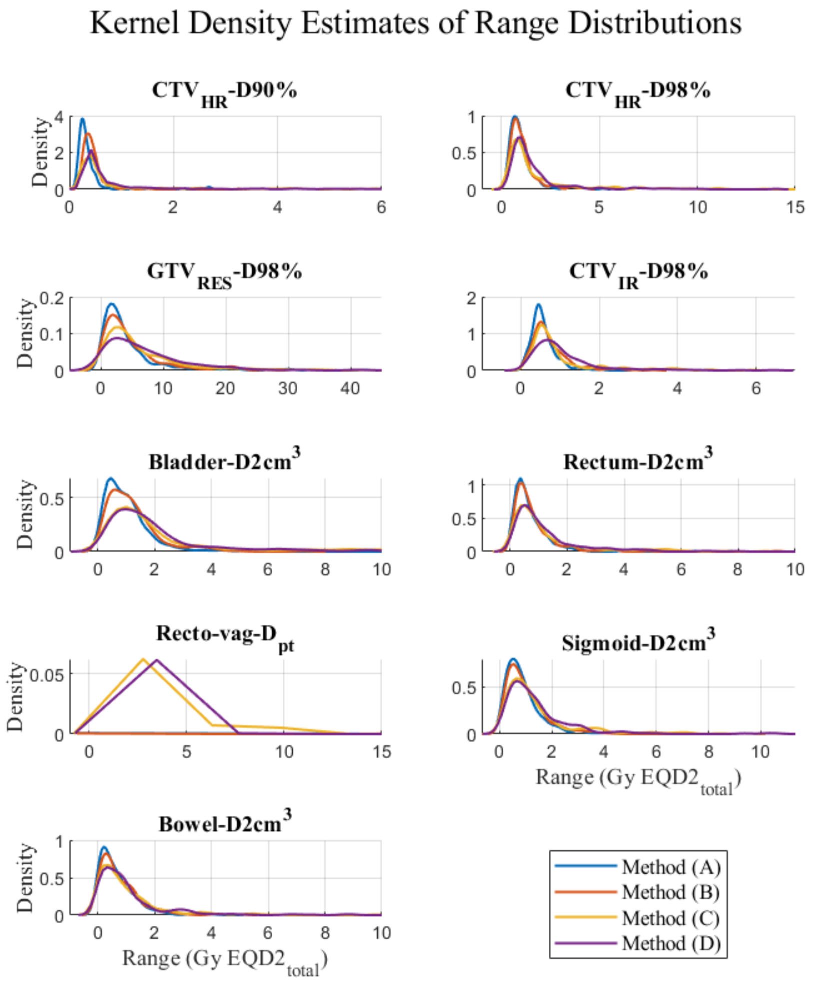


**Supplementary Figure 4:** Ranges (= maximum - minimum achieved value in 30 runs) over all patient cases, plotted as kernel density estimates, separately for the different DV metric values, for the plan according to $p = \text{argmax}_{p \in plans} [\min(\text{LCI}(p), \text{LSI}(p))]$, every color representing one method.

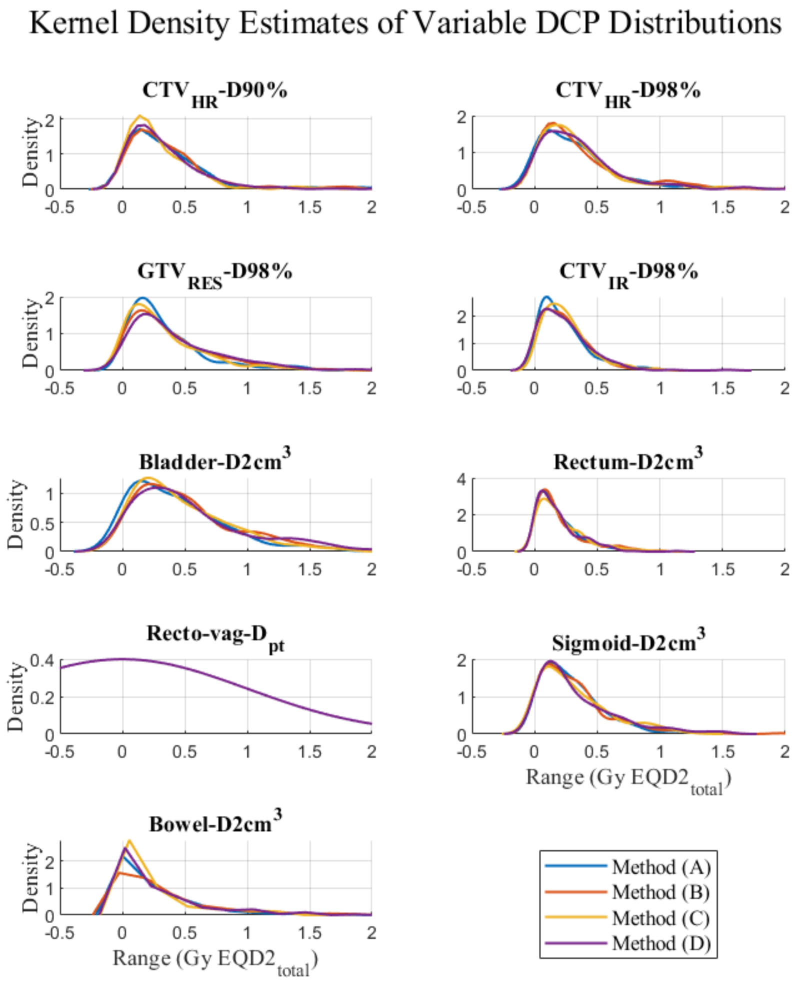


**Supplementary Figure 5:** Absolute value of difference when re-evaluating the plan according to $p = \text{argmax}_{p \in plans}[\min(\text{LCI}(p), \text{LSI}(p))]$, on a different set of 500,000 dose calculation points (DCP), over all patient cases, for one run per patient case. Plotted as kernel density estimates, separately for the different DV metric values, every color representing one method.

## D. Dosimetric comparison with clinically used plans

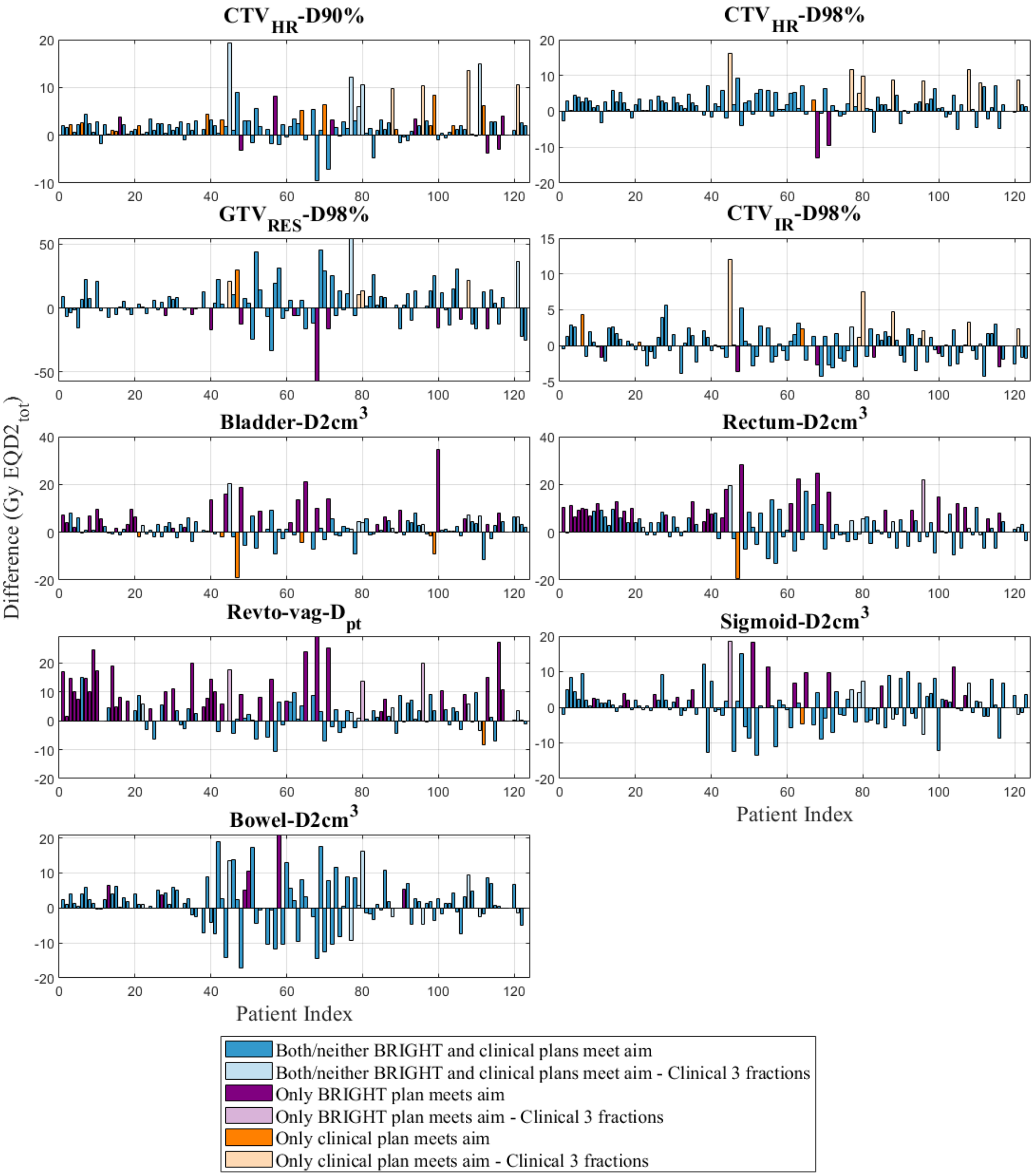


**Supplementary Figure 6:** Per-patient comparison between a BRIGHT plan (method (A)) and the clinical plan per DV metric. Values are calculated as total EQD2 over all fractions, with $EQD2_{clinical} - EQD2_{BRIGHT}$. Colors reflect whether each aim (not limit) is achieved according to the EMBRACE-II protocol. Some patients have been treated with 3 fractions clinically (marked in lighter colors), while all BRIGHT plans have been optimized for 4 fractions. For the BRIGHT plan, the plan with the most equally balanced coverage-sparing trade-off is chosen from each set of plans, implying that for challenging patients, a higher sparing than the clinical plan is automatically picked. The median BRIGHT values over 30 runs are taken. Patients for which not all fractions were available are not plotted.

## E. Statistics on catheter contribution

| | | median | std | minimum | maximum |
|---|---|---|---|---|---|
| Intrauterine | BRIGHT plans | 40.03% | 13.88% | 0.0% | 83.4% |
| | Clinical plans | 44.67% | 11.24% | 9.85% | 80.86% |
| Ovoids | BRIGHT plans | 32.47% | 14.83% | 0.0% | 74.04% |
| | Clinical plans | 38.90% | 10.07% | 0.0% | 63.79% |
| Needles | BRIGHT plans | 26.88% | 12.48% | 0.0% | 40.01% |
| | Clinical plans | 14.74% | 11.20% | 0.0% | 56.32% |

**Supplementary Table 1:** Statistics (median, standard deviation (std), minimum, maximum) on BRIGHT plans and clinical plans regarding contributions from the intrauterine part of the applicator, the ovoids, and the needles, for method (A). Values from the BRIGHT plans are taken from the plan with the most equally balanced coverage-sparing trade-off in each set of plans, and corresponds to the median over 30 runs.

## F. DV values corresponding to plans presented in Figure 6

| DV metric | | $D^{CTV_{HR}}_{90\%}$ | $D^{CTV_{HR}}_{98\%}$ | $D^{GTV_{res}}_{98\%}$ | $D^{CTV_{IR}}_{98\%}$ | $D^{A\text{-right}}_{point}$ | $D^{A\text{-left}}_{point}$ | $D^{Bladder}_{2cc}$ | $D^{Rectum}_{2cc}$ | $D^{Recto\text{-}vag}_{point}$ | $D^{Sigmoid}_{2cc}$ | $D^{Bowel}_{2cc}$ |
|---|---|---|---|---|---|---|---|---|---|---|---|---|
| (A) Baseline 3-obj. | coverage | 96.4 | 86.1 | 114.7 | 65.2 | 67.9 | 72.3 | 68.3 | 47.6 | 52.1 | 62.8 | 47.8 |
| | trade-off | 92.2 | 83.3 | 110.0 | 64.0 | 67.0 | 70.1 | 67.1 | 47.4 | 51.6 | 60.8 | 47.6 |
| | sparing | 90.1 | 80.9 | 105.6 | 62.7 | 65.5 | 69.1 | 64.9 | 47.1 | 50.9 | 60.0 | 47.3 |
| (B) Pear-shaped ROI | coverage | 96.3 | 86.1 | 117.8 | 65.2 | 67.7 | 71.4 | 70.3 | 47.6 | 52.2 | 63.1 | 47.9 |
| | trade-off | 92.2 | 82.9 | 112.4 | 63.8 | 66.1 | 69.6 | 68.5 | 47.4 | 51.5 | 61.5 | 47.7 |
| | sparing | 90.5 | 80.9 | 107.2 | 62.8 | 65.1 | 68.8 | 66.4 | 47.2 | 51.1 | 60.5 | 47.4 |
| (C) Contig. vol. | coverage | 96.2 | 85.7 | 116.9 | 65.0 | 67.3 | 71.3 | 70.5 | 47.6 | 52.2 | 63.1 | 48.1 |
| | trade-off | 92.3 | 82.5 | 108.6 | 63.4 | 67.0 | 69.8 | 67.1 | 47.3 | 51.4 | 61.3 | 47.7 |
| | sparing | 90.4 | 80.7 | 107.0 | 62.6 | 64.7 | 67.6 | 66.3 | 47.1 | 51.0 | 60.3 | 47.3 |
| (D) Contig. vol. + core-$CTV_{HR}$ | coverage | 96.4 | 85.1 | 127.0 | 64.6 | 66.2 | 70.1 | 68.2 | 47.6 | 52.1 | 63.2 | 47.9 |
| | trade-off | 92.3 | 81.7 | 122.5 | 63.0 | 64.3 | 68.3 | 68.0 | 47.2 | 51.4 | 61.3 | 47.5 |
| | sparing | 90.3 | 79.3 | 115.6 | 62.0 | 63.0 | 66.4 | 65.1 | 47.0 | 50.9 | 60.2 | 47.2 |

**Supplementary Table 2:** DV metric values for the plans presented in Figure 6: a high coverage, an equally balanced coverage-sparing trade-off, and a high sparing plan, for the four different options. Values correspond to total (EBRT + BT) EQD2 (in Gy). All values satisfy the aims from the EMBRACE-II protocol (except for the A points which were not included in the optimization).

## G. Comparison tri- vs. bi-objective approaches

The previously developed adaptive bi-objective approach[16] is compared to the tri-objective one explained in this work by comparing obtained dose volume (DV) values, as well as necessary runtimes.

In Supplementary Figure 7 an overview is given of the obtained results for the DV metrics from the EMBRACE-II protocol as well as for the added aims. The obtained DV values for the targets - which should be maximized (except for the $CTV_{HR}$ $D_{90\%}$ which has a minimization dose aim too) - are higher for the tri-objective than for the bi-objective method. OAR DV values - which should be minimized -, as well as the added DVH aims, are found to be comparable for both methods. Results from the Holm-Bonferroni-corrected Wilcoxon signed rank test are given in Supplementary Table 3 and indicate that a significant difference is found for 7/9 DV metrics.

The maximum runtime on an NVIDIA RTX A6000 GPU for the bi-objective method after which both convergence conditions (4) and (5) are satisfied is 10.6 min. The needed runtime for the tri-objective method was found to be 2.8 min (see section IV.C. in main paper), which is therefore substantially lower. Since brachytherapy patients are sedated and awaiting treatment while the treatment planning is carried out, keeping runtimes as low as possible is an important aspect. It is furthermore worth noting that these runtimes emanate from optimization on 20.000 dose calculation points per ROI, which leads to a negligible fallback of the Pareto approximation front when re-evaluating the results on 50.000 per ROI[9,16]. However, this many dose calculation points may not be needed for clinical use, which would then reduce the necessary total runtime considerably.

In conclusion, since obtained DV values are comparable between both methods, obtained values are even slightly higher for the tri-objective method, and more importantly, since the necessary runtime decreases substantially from the bi- to the tri-objective method, we retain the tri-objective method as the method of choice. Furthermore, treatment plans can more intuitively be represented in the tri-objective method, since the first two objectives include solely DV values from the EMBRACE-II protocol. It is therefore easily identifiable whether each plan satisfies these aims or not.

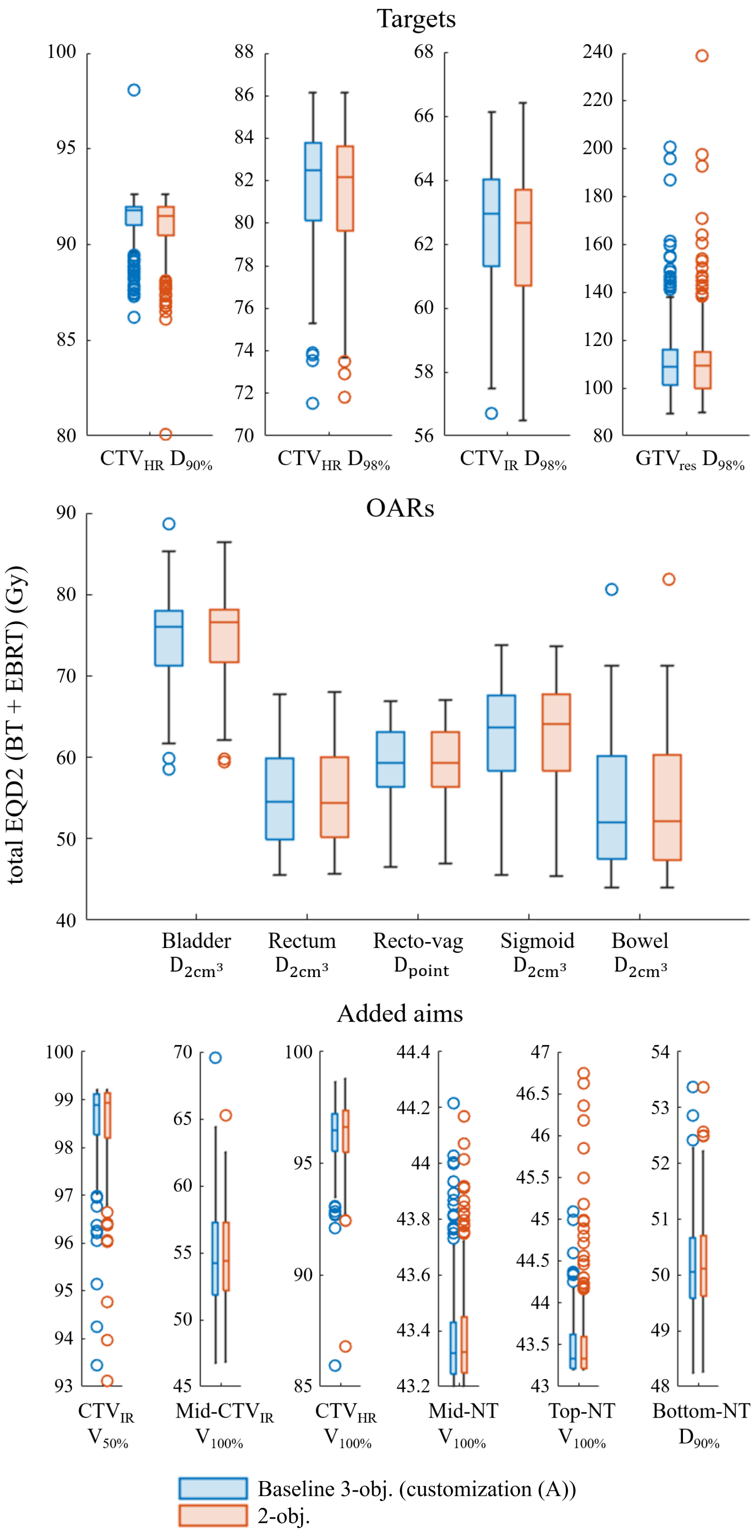


**Supplementary Figure 7:** Boxplots of obtained values for the DV metric aims from the EMBRACE-II protocol, as well as added aims, for the newly developed tri-objective approach (blue) compared with the previously published bi-objective approach (red). Every datapoint is one patient case (269 cases in total), corresponding to the median over 30 runs. The plan with the most equally balanced coverage-sparing trade-off is chosen from each set of plans. Abbreviation: NT: normal-tissue.

| DV metric | significance |
|---|---|
| $D_{90\%}^{\mathrm{CTV_{HR}}}$ | yes (p=0.000, $\alpha$=0.006) |
| $D_{98\%}^{\mathrm{CTV_{HR}}}$ | yes (p=0.000, $\alpha$=0.008) |
| $D_{98\%}^{\mathrm{GTV_{res}}}$ | yes (p=0.000, $\alpha$=0.020) |
| $D_{98\%}^{\mathrm{CTV_{IR}}}$ | yes (p=0.000, $\alpha$=0.007) |
| $D_{2cc}^{\mathrm{Bladder}}$ | yes (p=0.000, $\alpha$=0.006) |
| $D_{2cc}^{\mathrm{Rectum}}$ | yes (p=0.001, $\alpha$=0.050) |
| $D_{\mathrm{point}}^{\mathrm{Recto-vag}}$ | yes (p=0.000, $\alpha$=0.030) |
| $D_{2cc}^{\mathrm{Sigmoid}}$ | yes (p=0.000, $\alpha$=0.010) |
| $D_{2cc}^{\mathrm{Bowel}}$ | yes (p=0.000, $\alpha$=0.010) |

**Supplementary Table 3:** Results of Wilcoxon signed rank test (with Holm-Bonferroni-corrected $\alpha$ values) for bi- versus tri-objective method, where 'yes' indicates a significant difference.

## H. Customization selection by a specific institution

Two radiation oncologists, a medical physicist, and a radiation therapy technologist from Leiden University Medical Center were presented with plans resulting from optimization with the four different customizations. For six patient cases, they were asked to select the customization(s) that they prefer. Patient cases for which visually the largest differences in resulting 3D dose distributions were observed, were selected to this end. For the majority of the other cases, visual inspection indicated that similar 3D dose distributions were obtained for the four investigated customizations. The preferred customization(s) was/were selected by first presenting the medical team with the plan with the mathematically most balanced coverage-sparing trade-off (see Equation (5) in main paper). They could then scroll through the set of plans in order to inspect other plans from the front, should they wish to.

Supplementary Table 4 shows the preferred customization(s) picked per patient case, and indicates that even though for some patient cases two customizations were chosen, customization (D) was chosen for all cases and is therefore retained as the selected customization. This was mostly due to the shape of the dose distribution, being more centered to the target center and thereby leading to evident coverage of, e.g., the $\mathrm{GTV_{res}}$.

| Patient case | customization (A) | customization (B) | customization (C) | customization (D) |
|---|---|---|---|---|
| 1 | - | ✓ | - | ✓ |
| 2 | - | ✓ | - | ✓ |
| 3 | - | - | ✓ | ✓ |
| 4 | - | - | - | ✓ |
| 5 | - | - | - | ✓ |
| 6 | - | - | - | ✓ |

**Supplementary Table 4:** Preferred customization(s) by the Leiden University Medical Center for six patient cases.
